# One-step CRISPR-Cas9 protocol for the generation of plug & play conditional knockouts in *Drosophila melanogaster*

**DOI:** 10.1016/j.xpro.2021.100560

**Published:** 2021-05-25

**Authors:** Joyce J.S. Yu, Jean-Paul Vincent, Ian J. McGough

**Affiliations:** 1 The Francis Crick Institute, London NW1 1AT, UK

**Keywords:** CRISPR, Genetics, Model Organisms

## Abstract

This one-step method generates, for any locus, a conditional knockout allele in *Drosophila*. The allele carries a bright fluorescent marker for easy screening and an *attP* landing site for subsequent genetic manipulations. After removing the selectable marker with Cre, the *attP* site can be used to insert DNA fragments expressing tagged or mutant alleles to determine protein localization and function in a tissue- and stage-specific manner. Only a single round of CRISPR-Cas9-mediated editing is required.

For complete details on the use and execution of this protocol, please refer to the *DWnt4[cKO]* example in Yu et al. (2020).

## Highlights

• One-step CRISPR-Cas9 protocol to generate conditional knockout alleles in Drosophila
• Facile screening with a removeable fluorescent marker
• Integrase site included to enable subsequent “plug&play” manipulations

## Graphical abstract

**Figure:**
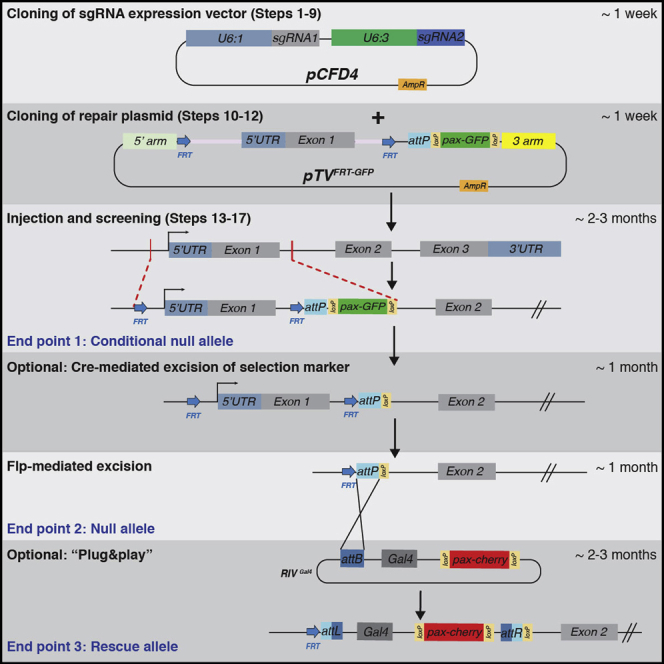

## Before you begin

### sgRNA selection

**Timing: 1 h**

**Figure 1:**
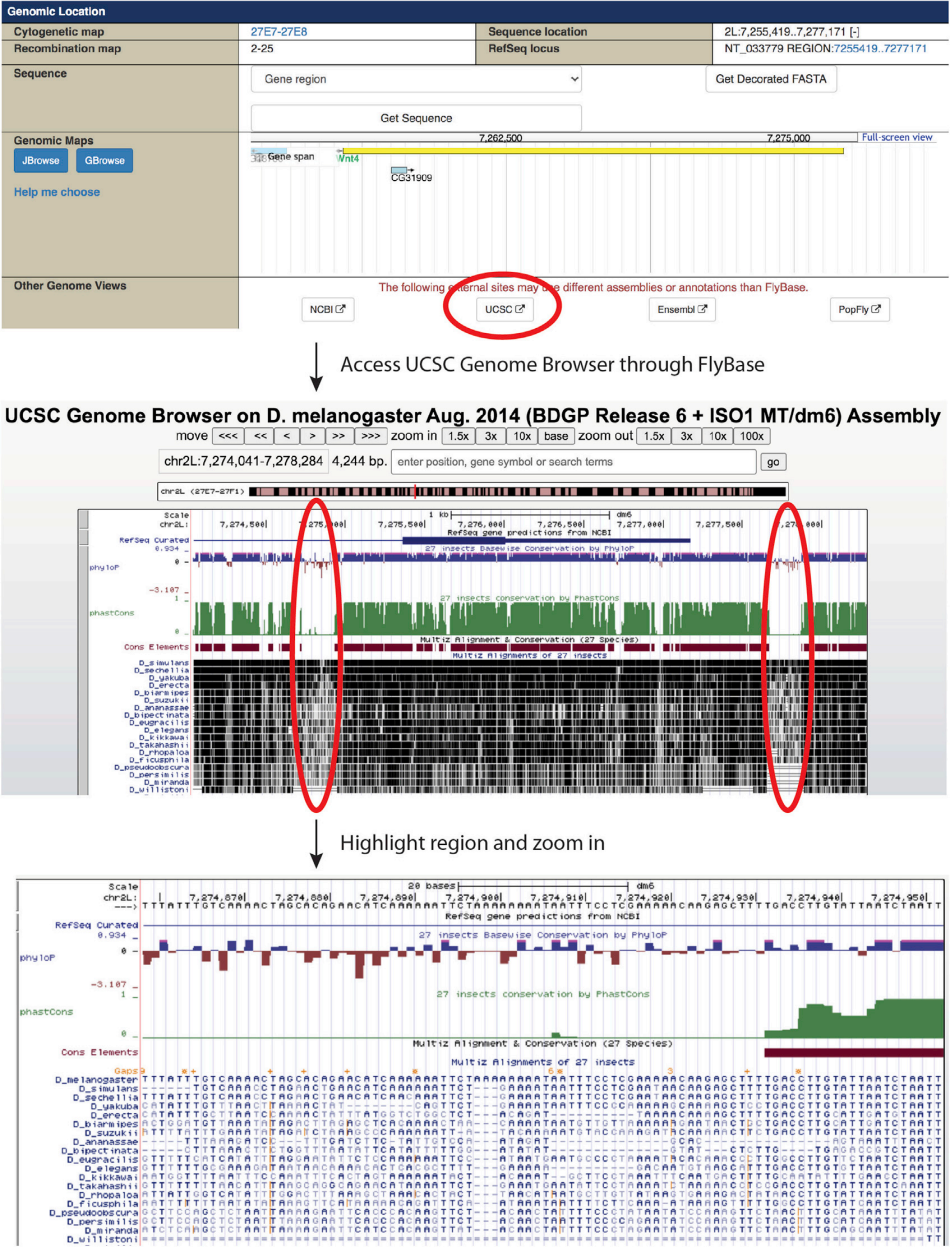
Screenshots of how to identify non-conserved regions using the UCSC Genome Browser Example of *DWnt4*, showing non-conserved regions upstream of the 5′UTR and downstream of the 1^st^ exon. The non-conserved region can be highlighted and zoomed in to obtain the DNA sequence. Note that *DWnt4* is transcribed from the reverse strand.

Select sgRNAs and order oligonucleotides for insertion of corresponding coding DNA into the *pCFD4* vector (Addgene: 49411), which allows tandem gRNA expression (Port et al., 2014). Target sites should be chosen such that they direct Cas9 to the 5′ and 3′ ends of the genomic sequence to be deleted. In the example for *DWnt4* in Figure 1, these are located slightly upstream of the 5′UTR and downstream of the 1^st^ exon.

***Alternatives:*** An alternative is the newer *pCFD5* vector (Addgene: 73914), which allows the efficient expression of up to 6 sgRNAs (Port and Bullock, 2016).

1. Obtain the full genomic sequence of your gene of interest from FlyBase (https://flybase.org/) by searching the gene name and label all coding exons and UTRs.

***Note:*** Applications such as SerialCloner (http://serialbasics.free.fr/Serial_Cloner.html) or SnapGene (https://www.snapgene.com/) provide user-friendly means of displaying and annotating genomic sequences.

2. On the FlyBase page for the gene of interest, view the genomic sequence on the UCSC genome browser (http://genome-euro.ucsc.edu/) (Figure 1).
  a. Identify and highlight a non-conserved region upstream of the region to be deleted (in this instance the 5′UTR and 1^st^ exon of DWnt4 is to be deleted, and the chosen non-conserved region is approximately 500 base pairs upstream of the 5′UTR).
  b. Identify and highlight a second non-conserved region downstream of the region to be deleted (in this instance the chosen non-conserved region is approximately 500 base pairs downstream of the 1^st^ exon).

**CRITICAL:** The presence of residual sequences from FRT, attP or loxP sites could impair expression of your gene of interest if they are located in important (conserved) regulatory regions. Non-conserved areas are chosen to maximise the chance that reintegrated DNA is faithfully expressed.

3. For each non-conserved region from steps 2a-b, copy and paste the sequence into a CRISPR target finder tool such as http://targetfinder.flycrispr.neuro.brown.edu/. Select the “*Drosophila melanogaster*” genome and the guide length as 20nt. On the next page, select ‘High Stringency’, and ‘NGG only’ for PAM.
4. Select sgRNAs that have no predicted off-target sites.

**CRITICAL:** if off-target sites are unavoidable, choose sgRNAs with predicted off-target sites that are on a different chromosome from the gene of interest. This will allow unintended modifications to be more easily crossed out subsequently (see troubleshooting 1).

### sgRNA primer design

**Timing: 3 days**

**Figure 2:**
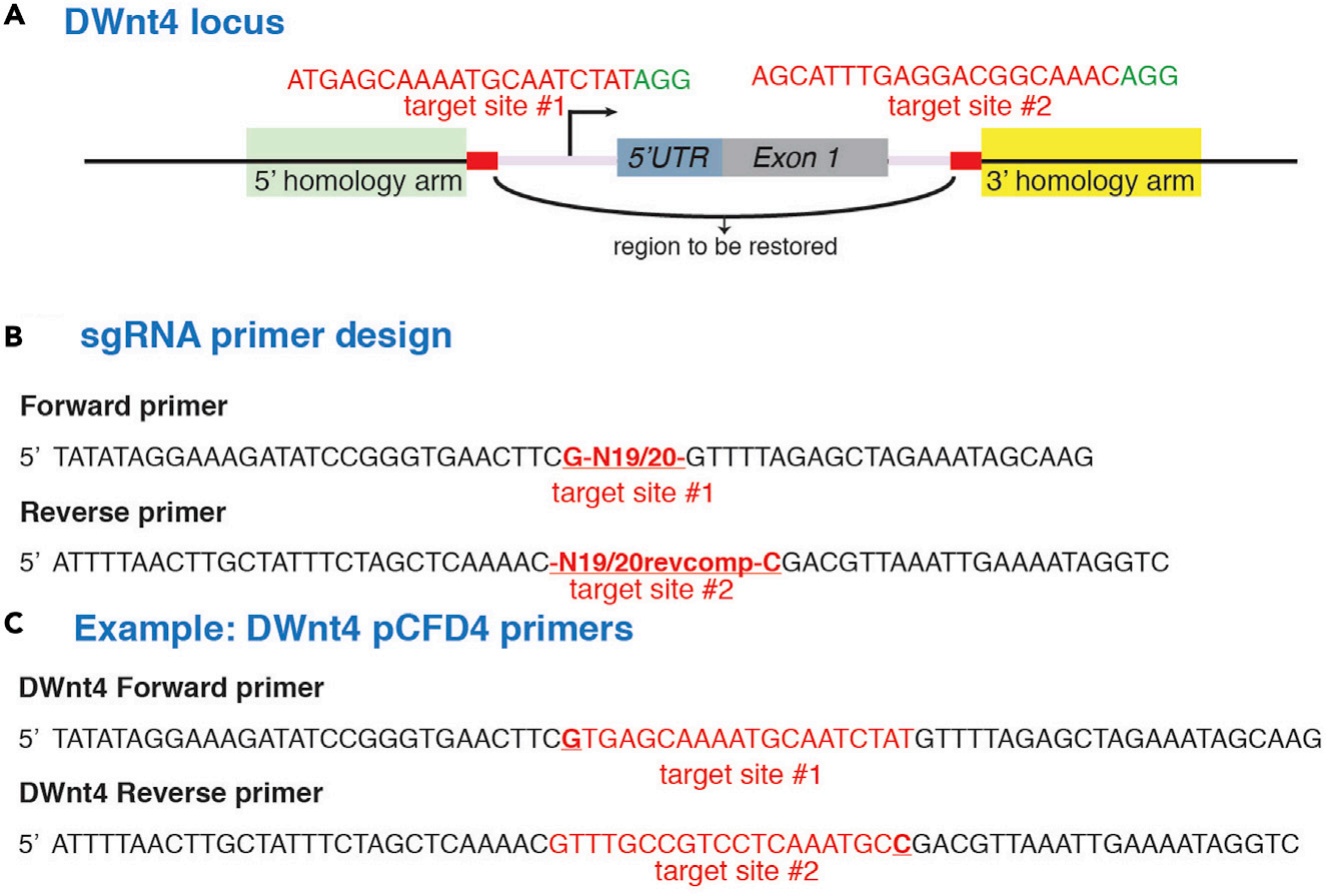
sgRNA primer design (A) Locus of *DWnt4*, showing locations of the two Cas9 target sites, which flank the 5′UTR and first exon. A repair plasmid illustrated in Figure 3 will be used to re-introduce the intervening sequence flanked by FRT sites. (B) Primer design for cloning the target site sequences into the *pCFD4* gRNA expression vector (Port et al., 2014). (C) Primers for the DWnt4 *pCFD4* cloning example.

To ensure that no SNP is present in the chosen target sequences, which could prevent efficient DNA cleavage, the regions around the two chosen sgRNA target sites are sequenced from the genomic DNA obtained from the injection host strain (Figure 2A).

***Note:*** For commonly used Cas9 lines, it is now possible to search for target sites within the sequenced Cas9 genomes, thus eliminating the need to perform step 5. Examples of these tools include https://www.flyrnai.org/crispr3/ and http://targetfinder.flycrispr.neuro.brown.edu/. If not using such a sequenced Cas9 line proceed with step 5.

5. Design primers for PCR amplification of ∼150bp on either side of the two chosen sgRNA target sites. PCR from genomic DNA obtained from injection host strain (Figure 2A).
  a. The host strain should bear a Cas9-expressing transgene on a different chromosome from that of the target locus. This allows for subsequent removal of the Cas9 -expressing transgene and its associated markers. A list of available Cas9-expressing strains can be found in (Kondo and Ueda, 2013) and at websites such as, flyCRISPR website (https://flycrispr.org/reagents/), and the CRISPR fly design website (http://www.crisprflydesign.org/flies/). Lines in which Cas9 is driven by germline promoters such as vasa-Cas9 or nanos-Cas9 should be used. This avoids somatic alteration, which could affect the survival or fitness of the organism. In the case of the *DWnt4[cKO]* example, *yw;;nos-Cas9(III-attP2)* from (Kondo and Ueda, 2013) was used.
    **CRITICAL:** In certain Cas9 lines, the Cas9-expressing transgene is marked with GFP, which would interfere with the screening via the pax-GFP marker later on. Avoid using such Cas9 lines.
  b. In preparation for extraction of genomic DNA, pick ∼5 individuals and immobilize flies from the Cas9 -expressing strain by placing them at 4°C for 5 min.
  c. Crush the flies with a pestle in an Eppendorf tube and follow the ‘Single fly DNA prep for PCR’ protocol found at http://francois.schweisguth.free.fr/protocols/Single_fly_DNA_prep.pdf.
    ***Note:*** If the gDNA quality is of insufficient purity or yield we recommend gDNA be extracted with the ChargeSwitch gDNA Micro Tissue Kit (Invitrogen), following the manufacturer’s protocol (https://www.thermofisher.com/document-connect/document-connect.html?url=https%3A%2F%2Fassets.thermofisher.com%2FTFS-Assets%2FLSG%2Fmanuals%2Fchargeswitch_gDNA_tissue.QRC.pdf&title=Q2hhcmdlU3dpdGNoIGdETkEgVGlzc3VlIEtpdHMgUVJD). We have observed gDNA extracted with this kit also provides a better template for amplification of larger DNA fragments (such as during the cloning of homology repair arms in steps 10 and 11 Repair Plasmid Cloning). As the gDNA kit is designed for tissues from larger organisms, half the recommended volume of buffers and solutions suffices.
  d. To PCR amplify the two regions containing the target sites from the gDNA, set up the following 50uL PCR reaction mix.

**Table:** 

Reagent	Final concentration	Amount
Q5 2× Master Mix	1×	25 μL
Forward Primer, 10 μM	0.5 μM	2.5 μL
Reverse Primer, 10 μM	0.5 μM	2.5 μL
gDNA	n/a	2 μL
ddH_2_O	n/a	To 50 μL
  e. Follow the PCR cycling conditions according to the protocol for Q5 2× Master Mix (https://international.neb.com/protocols/2012/12/07/protocol-for-q5-high-fidelity-2x-master-mix-m0492).

**Table:** 

PCR cycling conditions			
Steps	Temperature	Time	Cycles
Initial Denaturation	98°C	30 s	1
Denaturation	98°C	10 s	35–40 cycles
Annealing	58°C–67°C (see Note)	30 s	
Extension	72°C	30 s/kb	
Final extension	72°C	2 min	1
Hold	4°C–10°C	Forever

    ***Note:*** The annealing temperature will depend on the primers used. Determine the suitable annealing temperature using NEB Tm calculator: https://tmcalculator.neb.com/
  f. Run the PCR reaction mix in 1% agarose gel, purify the PCR product with the QIAquick Gel Extraction Kit, according to the manufacturer’s instructions (https://www.qiagen.com/gb/resources/resourcedetail?id=a72e2c07-7816-436f-b920-98a0ede5159a&lang=en).
  g. Send the purified PCR product for sequencing and align the resulting sequence with the published sequence on FlyBase.
    ***Note:*** If SNPs are present in either of the target sites, return to the previous section ‘sgRNA selection’ and choose alternative target site(s).

6. Primers for cloning sgRNA sequences into the *pCFD4* vector are designed according to Port et al., 2014 (Figure 2B). Primers for the DWnt4 pCFD4 cloning are shown in Figure 2C.

**CRITICAL:** If the protospacer sequence of your chosen target site starts with a G then N will be 19. If it does not start with a G enter all 20 nucleotides. Alternatively, as in the example in Figure 2C, if the 1^st^ nucleotide is not a G, substitute the 1^st^ nucleotide with a G, such that the sequence becomes G+19 nucleotides.

### Repair plasmid and primer design

**Timing: 1 h**

The parent repair plasmid, *pTV*^*GFP*^ was derived from *TV*^*ΔattP*^*-Pax-Cherry*, where the pax-Cherry marker is replaced with pax-GFP (Poernbacher et al., 2019, Yu et al., 2020). The pax-GFP marker allows selection based on GFP signal in the larval CNS, as well as the adult eye and ocelli (Horn et al., 2000). The boundaries of the 5′ and 3′ homology arms should be no more than 50bp away from the double strand break, ideally less than 10bp away if possible. Crucially, in order to prevent Cas9 from cleaving the repair plasmid before it has a chance to integrate, the homology arms MUST NOT contain the full target site. The homology arms may contain part of the target site but MUST NOT include the PAM sequence (nGG). Alternatively, a few bases in the 10 nucleotides proceeding the PAM and the PAM itself can be changed in the target site sequence in the repair plasmid (as in the example of *DWnt4[cKO]*). The safest strategy is to include no more than the first 5–10bp of the target site in your homology arm.

**Figure 3:**
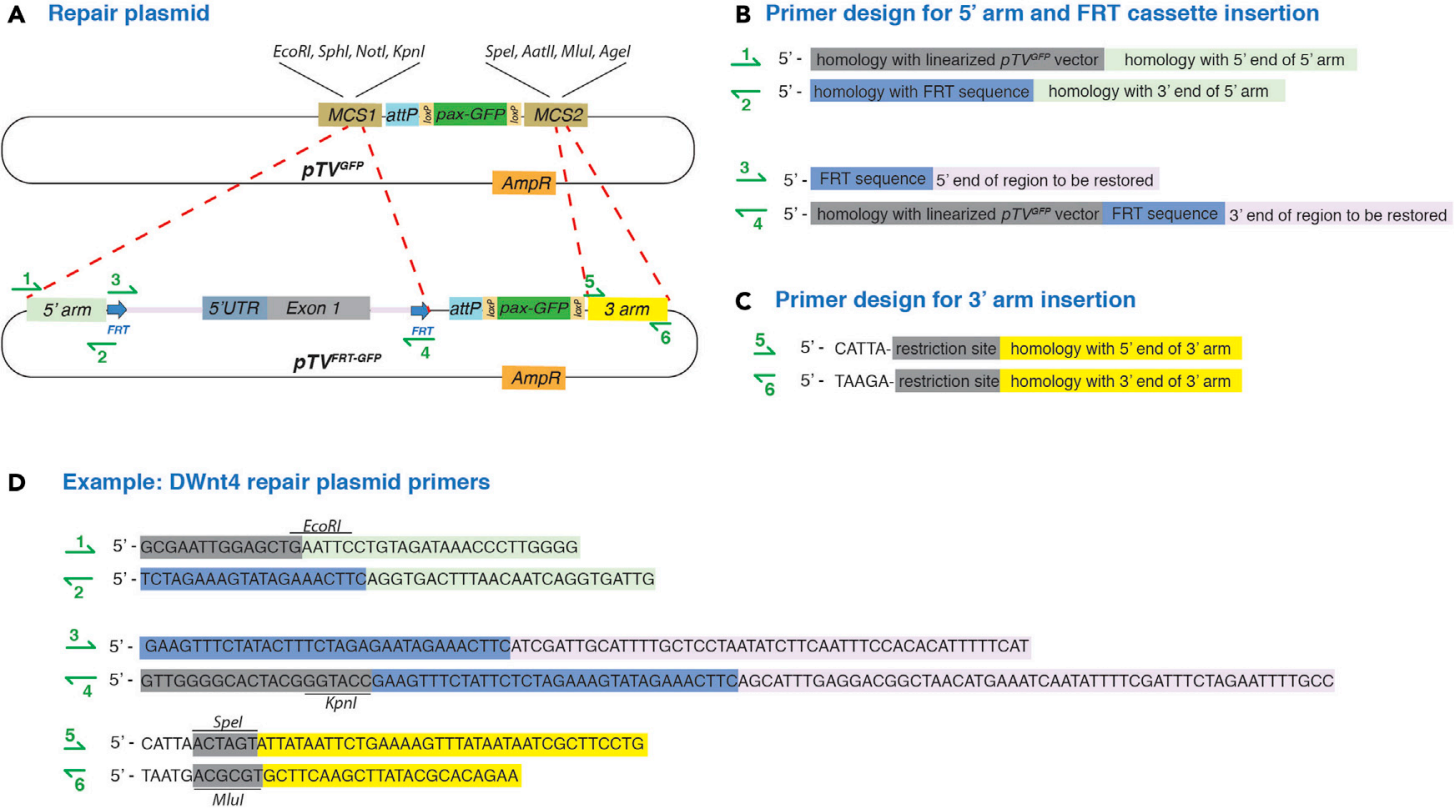
Repair plasmid primer design (A) Construction of *pTV*^*FRT-GFP*^ (repair plasmid) from the *pTV*^*GFP*^ parent plasmid. Locations of the primers required to insert the 5′ homology arm and FRT cassette are shown. The 5′ and 3′ homology arms overlap with half of the target sites, in order to prevent Cas9 from cleaving the repair plasmid before it has a chance to integrate. (B) Components that make up primers 1–4 for insertion of the 5′ arm and FRT cassette. (C) Components that make up primers 5–6 for insertion of the 3′ arm. (D) Example of primers for generating the DWnt4 repair plasmid.
  c. Design and order primers to PCR amplify the 3′ homology arm, including the chosen restriction sites at the 5′ ends of the primers to facilitate cloning into the *pTV*^*GFP*^ *vector* (primers 5–6 in Figure 3C).
8. Design primers for insertion of the 5′ homology arm and the FRT cassette into multiple cloning site 1 (MCS1) using Gibson Assembly (NEB):
  a. Identify ∼1000bp upstream of CRISPR-Cas9 target site number 1 (starting from the middle of the site. This will be the 5′ homology arm (Figure 2A).
  b. Select restriction sites that are only present in the MCS1, making sure the sites are not present in the 3′arm (Figure 3A).
  c. Design and order primers to PCR amplify the 5′ homology arm, including a segment homologous to the linearized vector at the beginning of the forward primer (primers 1 and 2 in Figure 3B).
  d. Design and order primers to PCR amplify the region between the two target sites, nesting the FRT sequence within both the forward and the reverse primers (FRT71 sequence was used in the example of DWnt4[cKO]: GAAGTTCCTATTCCGAAGTTCCTATTCTCTAGAAAGTATAGGAACTTC) (primers 3 and 4 in Figure 3B).

***Note:*** Steps 7 and 8 can be swapped around, depending on the availability of unique restriction sites.

7. Design primers for insertion of the 3′ homology arm into the *pTV*^*GFP*^ repair vector via multiple cloning site 2 (MCS2) using T4 ligation (NEB):
  a. Identify ∼1000bp downstream of CRISPR-Cas9 target site number 2 (starting from the middle of the site. This will be the 3′ homology arm (Figure 2A).
  b. Select two restriction sites that are only present in the MCS2 (Figure 3A) (see troubleshooting 2)

***Alternatives:*** A more commonly used FRT sequence can be used instead (sequence: GAAGTTCCTATTCTCTAGAAAGTATAGGAACTTC). FRT71 was used in the *DWnt4[cKO]* example because this cKO was made in a background of another conditional allele that already contains FRT sites.

## Key resources table

**Table:** 

REAGENT or RESOURCE	SOURCE	IDENTIFIER
**Bacterial and virus strains**		

DH5α competent cells	Thermo Fisher	Cat # 18265017

**Chemicals, peptides, and recombinant proteins**		

Agarose	Sigma-Aldrich	Cat # 05066
Proteinase K	Thermo Fisher	Cat # EO0491
Tris-HCl	Sigma-Aldrich	Cat # 10812846001
NaCl	Sigma-Aldrich	Cat # S9888
EDTA	Sigma-Aldrich	Cat # E9884
CutSmart buffer, 10×	NEB	Cat #B7204
Ampicillin	Sigma-Aldrich	Cat # A0166
Yeast extract	Oxoid	Cat# LP0021
Tryptone	Oxoid	Cat# LP0042
Agar powder	Sigma-Aldrich	Cat# 05040
Gibson Assembly Master Mix	NEB	Cat # E2611
Q5 High-Fidelity 2× master mix	NEB	Cat # M0492
T4 DNA ligase	NEB	Cat # M0202
T4 DNA ligase reaction buffer	NEB	Cat # B0202
BbsI-HF	NEB	Cat # R3539

**Critical commercial assays**		

ChargeSwitch gDNA Micro Tissue Kit	Invitrogen	Cat # CS11203
QIAquick Gel Extraction Kit	QIAGEN	Cat # 28704
QIAprep Spin Miniprep Kit	QIAGEN	Cat # 27104
Invitrogen PureLink HiPure Plasmid Maxiprep Kit	Invitrogen	Cat # K210006

**Experimental models: Organisms/ strains**		

*Drosophila melanogaster : yw;;nos-Cas9(III-attP2)*	(Kondo and Ueda, 2013)	N/A
*Drosophila melanogaster : w1118*	Crick Fly Facility	N/A

**Oligonucleotides**		

FRT: GAAGTTCCTATTCTCTAGAAAGTATAGGAACTTC	N/A	N/A
FRT71 : GAAGTTCCTATTCCGAAGTTCCTATTC TCTAGAAAGTATAGGAACTTC	N/A	N/A
*pCFD4* verification : GACACAGCGCGTACGTCCTTCG	N/A	N/A
*pTV*^*GFP*^ 5′arm forward primer for verification: CAGTCACGACGTTGTAAAACGA	N/A	N/A
*pTV*^*GFP*^ Pax reverse primer for verification: GAATTAGCTCTAATTGAATTAGTCTCTAATTGAATT	N/A	N/A
*pTV*^*GFP*^ 3′arm forward primer for verification: GTTGTGGTTTGTCCAAACTCAT	N/A	N/A
*pTV*^*GFP*^ 3′arm reverse primer for verification: CCATGATTACGCCAAGC	N/A	N/A

**Recombinant DNA**		

*pCFD4*	Port et al., 2014	Addgene: 49411
*pCFD5*	(Port and Bullock, 2016)	Addgene: 73914
*pTV*^*GFP*^	(Yu et al., 2020)	Addgene: 169445

**Software and algorithms**		

UCSC Genome Browser	(Kent et al., 2002)	https://genome.ucsc.edu/
FlyBase	n/a	https://flybase.org
Serial Cloner	n/a	http://serialbasics.free.fr/Serial_Cloner.html
Target Finder	(Gratz et al., 2014)	http://targetfinder.flycrispr.neuro.brown.edu/

## Materials and equipment

**Table:** Ampicillin LB plates

Reagent	Final concentration	Amount
Yeast extract	n/a	5 g
Tryptone	n/a	10 g
NaCl	n/a	10 g
ddH_2_O	n/a	To 1 L
Ampicillin	100 μg/mL	0.1 g
Agar powder	n/a	15 g
Total	n/a	1 L

LB buffer is mixed with the 15 g of agar powder and is autoclaved at 121^°^C for 15 min. Add Ampicillin after the solution is cooled to 55^°^C. Pour the buffer into petri dishes and allow them to solidify. The Ampicillin LB plates can be stored at 4^°^C for up to 2 months.

**Table:** Squishing buffer for single fly genomic DNA preparation

Reagent	Final concentration
Tris-HCL (pH=8.2)	10 mM
EDTA	1 mM
NaCl	25 mM
ddH_2_O	n/a
Proteinase K	200 μg/mL

Proteinase K should be added right before use. The squishing buffer can be stored at room temperature for up to one month.

## Step-by-step method details

### sgRNA cloning

**Timing: 3–4 days**

The protocol for cloning sgRNAs into the *pCFD4* vector is according to (Port et al., 2014), which can be found at http://www.crisprflydesign.org/wp-content/uploads/2014/06/Cloning-with-pCFD4.pdf. The protocol for the alternative *pCFD5* vector can be found at http://www.crisprflydesign.org/wp-content/uploads/2016/07/pCFD5cloningprotocol.pdf.

1. Using *pCFD4* vector as a template, run a PCR with the primers from Before you begin step 6
  a. Set up the following PCR reaction mix

**Table:** 

Reagent	Final concentration	Amount
Q5 2× Master Mix	1×	25 μL
Forward Primer, 10 μM	0.5 μM	2.5 μL
Reverse Primer, 10 μM	0.5 μM	2.5 μL
*pCFD4* vector	n/a	50 ng
ddH_2_O	n/a	To 50 μL
  b. Run the PCR by referring to the conditions from Before you begin step 5e. The optimal annealing temperature is 61^°^C.

2. Digest *pCFD4* vector with BbsI-HF at 37^°^C for 1 h, according to the following reaction mix.

**Table:** 

Reagent	Final concentration	Amount
*pCFD4* vector	n/a	2 μg
CutSmart buffer, 10×	1×	1 μL
BbsI-HF	n/a	0.5 μL
ddH_2_O	n/a	to 10 μL

3. Run PCR product and digested vector on 1% agarose gel.
  a. PCR product size: 600bp
  b. Digested vector size: 6.4 kb
4. Gel purify the PCR product and the digested vector with the QIAquick Gel Extraction Kit, according to the manufacturer’s instructions (https://www.qiagen.com/gb/resources/resourcedetail?id=a72e2c07-7816-436f-b920-98a0ede5159a&lang=en).
5. Insert the PCR product into the linearized vector using Gibson Assembly (NEB) according to the manufacturer’s instructions (https://international.neb.com/protocols/2012/12/11/gibson-assembly-protocol-e5510).
6. Transform with competent bacteria and plate on Ampicillin LB agar plates. The final concentration of Ampicillin is 100 μg/mL.
7. Pick a few individual colonies and culture 16 h for MiniPrep (Qiagen) the following day, following the manufacturer’s instructions (https://www.qiagen.com/us/resources/resourcedetail?id=331740ca-077f-4ddd-9e5a-2083f98eebd5&lang=en).
8. Verify insertion by sequencing with the following primer:
  a. Primer for verification: GACACAGCGCGTACGTCCTTCG
9. Purify the *pCFD4* plasmid with a Maxiprep kit (Invitrogen) following the manufacturer’s instructions (https://www.thermofisher.com/document-connect/document-connect.html?url=https%3A%2F%2Fassets.thermofisher.com%2FTFS-Assets%2FLSG%2Fmanuals%2Fpurelink_hipure_plasmid_qrc.pdf&title=UXVpY2sgUmVmZXJlbmNlOiBQdXJlTGluayBIaVB1cmUgUGxhc21pZCBETkEgUHVyaWZpY2F0aW9uIEtpdHM=).

**Pause point:** the *pCFD4* plasmid can be stored at −20^°^C for an extended period until injection.

### Repair plasmid cloning

**Timing: ∼ 1 week**

Components (5′ arm, region flanked by FRT sites, and 3′ arm) to be inserted into the parent repair vector *pTV*^*GFP*^ are PCR amplified from genomic DNA extracted from the selected Cas9 line (Before you begin step 5).

10. Inserting 3′ homology arm into MCS2:
  a. PCR amplify the fragment using primers designed from Before you begin step 7c. Refer to Before you begin step 5d for PCR reaction mix, and Before you begin step 5e for PCR condition. Gel purify the PCR product (refer to Before you begin step 5f).
  b. Digest *pTV*^*GFP*^ and the PCR product with the two restriction enzymes selected in Before you begin step 7b, setting up the following reaction mix for digesting the *pTV*^*GFP*^ and the PCR product respectively. The reaction conditions required for the selected restriction enzymes can be found using the NEB Double Digest Finder (http://nebcloner.neb.com/).
    Reaction mix for digesting *pTV*^*GFP*^ backbone:

**Table:** 

Reagent	Final concentration	Amount
*pTV*^*GFP*^	n/a	2 μg
CutSmart buffer, 10× (see Note)	1×	1 μL
Restriction enzyme 1	n/a	0.5 μL
Restriction enzyme 2	n/a	0.5 μL
ddH_2_O	n/a	to 10 μL

    ***Note:*** Other buffers may be required, depending on the restriction enzymes selected. Use NEB Double Digest Finder (http://nebcloner.neb.com/) to identify the suitable buffer.
    Reaction mix for digesting the PCR product:

**Table:** 

Reagent	Final concentration	Amount
PCR product	n/a	34 μL
CutSmart buffer, 10×	1×	4 μL
Restriction enzyme 1	n/a	1 μL
Restriction enzyme 2	n/a	1 μL

    ***Note:*** Other buffers may be required, depending on the restriction enzymes selected. Use NEB Double Digest Finder (http://nebcloner.neb.com/) to identify the suitable buffer.
  c. Ligate the digested *pTV*^*GFP*^ backbone and the PCR product, following the manufacturer’s protocol for T4 DNA Ligase (NEB) for reaction mix and conditions (https://international.neb.com/protocols/0001/01/01/dna-ligation-with-t4-dna-ligase-m0202). This produces the *pTV*^*GFP*^*-3*′*arm* vector.
  d. Transform with competent bacteria and plate on Ampicillin plates.
  e. Pick a few individual colonies and culture overnight for MiniPrep (Qiagen).
  f. Verify insertion by sequencing with the following primers:
    i. *pTV*^*GFP*^ 3′arm forward primer for verification: GTTGTGGTTTGTCCAAACTCAT
    ii. *pTV*^*GFP*^ 3′arm reverse primer for verification: CCATGATTACGCCAAGC

11. Inserting 5′ homology arm and FRT cassette into MCS1:
  a. PCR amplify the fragments using primers designed from Before you begin step 7c. Refer to Before you begin step 5d for PCR reaction mix, and Before you begin step 5e for PCR condition. Gel purify the PCR product (refer to Before you begin step 5f).
  b. Digest *pTV*^*GFP*^*-3*′*arm* with the two restriction enzymes selected in Before you begin step 8b, referring to step 10b for reaction mix and condition.
  c. Assemble using Gibson assembly according to the manufacturer’s protocol (https://international.neb.com/protocols/2012/12/11/gibson-assembly-protocol-e5510), with the digested *pTV*^*GFP*^*-3*′*arm* as the backbone and the two PCR products in step 10a as inserts. This generates the *pTV*^*FRT-GFP*^ donor plasmid (see troubleshooting 3).
  d. Transform with competent bacteria and plate on Ampicillin plates.
  e. Pick a few individual colonies and culture overnight for MiniPrep (Qiagen).
  f. Verify insertion by sequencing with the following primers:
    i. *pTV*^*GFP*^ 5′arm forward primer for verification: CAGTCACGACGTTGTAAAACGA
    ii. *pTV*^*GFP*^ Pax reverse primer for verification: GAATTAGCTCTAATTGAATTAGTCTCTAATTGAATT

***Note:*** Steps 10 and 11 can be swapped around, depending on the availability of unique restriction sites

***Note:*** Instead of Gibson assembly, the components can be sequentially cloned into the repair vector using restriction sites from MCS1 via ligation (see troubleshooting 3).

12. Purify the *pTV*^*FRT-GFP*^ plasmid with a Maxiprep kit (Invitrogen).

**Pause point:** the *pTV*^*FRT-GFP*^ plasmid can be stored at −20^°^C for an extended period until injection.

### Injection and screening

**Timing: ∼2 weeks**

**Figure 4:**
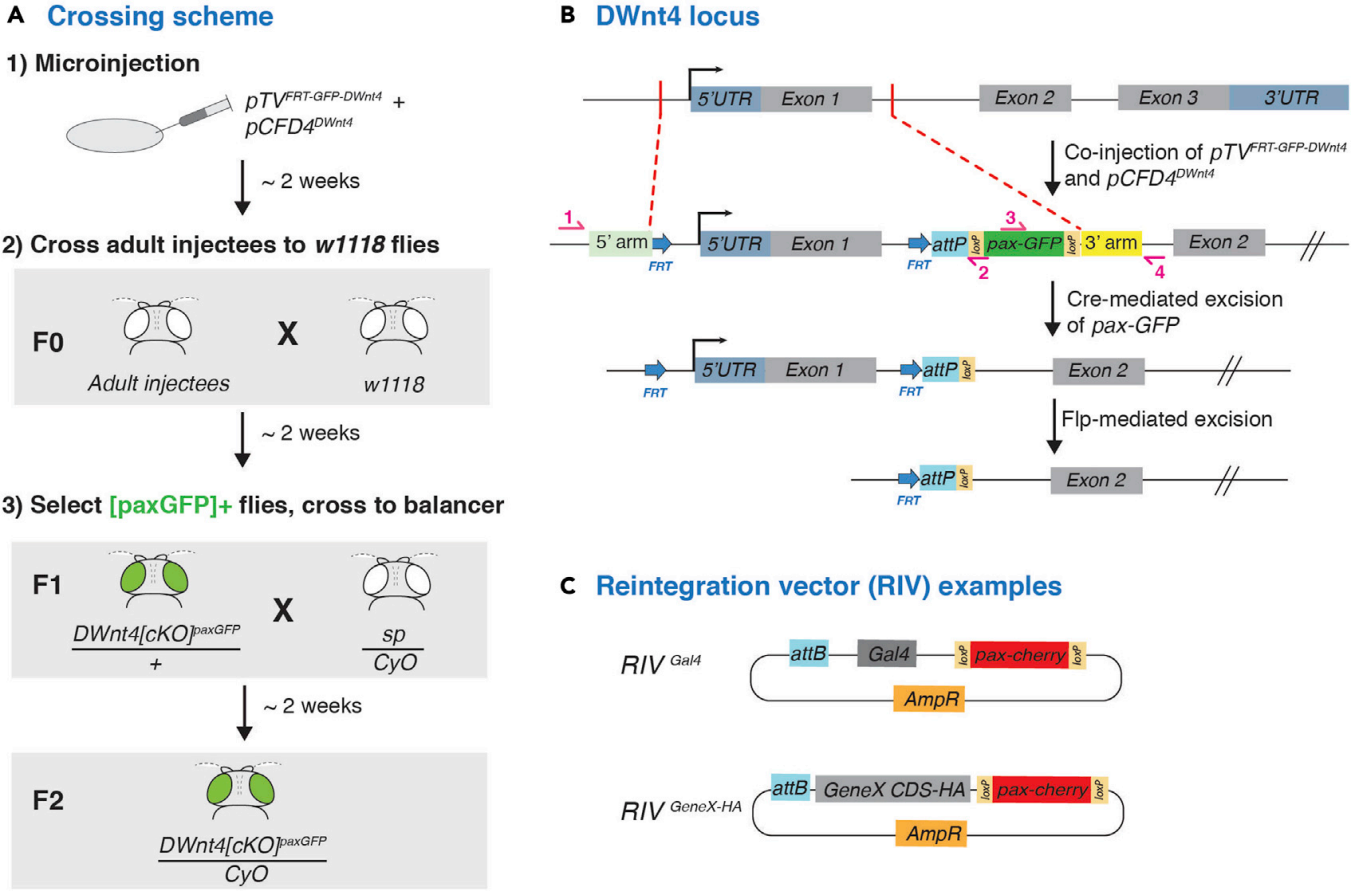
Generation of the *DWnt4[cKO]* line and possible subsequent steps (A) Crossing scheme after injection to establish the *DWnt4[cKO]* line. (B) Diagram of the steps to generate a conditional allele of *DWnt4*, showing replacement of the sequence between the two target sites with FRT-flanked rescue DNA and the excisable *pax-GFP* cassette. For the verification of correct insertion, the binding location of two pairs of primers (1 and 2, 3 and 4) are shown. (C) Examples of reintegration vectors, for subsequent introduction of DNA fragments into the locus via the attP site using PhiC31-mediated integration (Baena-Lopez et al., 2013).

The gRNA vector *pCFD4* can be co-injected with the *pTV*^*FRT-GFP*^ repair plasmid into embryos of any transgenic line that express Cas9 (Figure 4A). If in-house injection facilities are not available, the injection mix can be sent to companies for injection into commonly available Cas9 lines.

13. Expand the Cas9-expressing strain to be used for injection
14. The injection mix contains the *pCFD4* and the *pTV*^*FRT-GFP*^ plasmids in ddH_2_O.

***Note:*** Plasmid concentrations can vary. Recommended concentrations are 100 ng/μL for *pCFD4* and 500 ng/μL for *pTV*^*FRT-GFP*^, for injecting into 300–400 embryos. Injection volume is typically less than 5% of the egg volume.

***Note:*** Recommended amounts of each plasmid are 300 μg for *pCFD4* and 500 μg for *pTV*^*FRT-GFP*^, for injecting into 300–400 embryos.

**Timing: 1–2 months**

15. Injected embryos are grown to adulthood and crossed to *w1118* flies. Screen for GFP signal in the next generation larval CNS or the adult eyes/ocelli (Figure 4A).
16. Select individual transformants and establish balanced GFP-positive transformant lines (see troubleshooting 4 and 5) (Figure 4A).
17. Extract gDNA from transformant lines and PCR verify the insertion is in the correct location. Design two sets of primers for the PCR verification, such that one of each set binds just outside the regions that correspond to the 5′ and 3′ arms, and the other binds to the GFP coding sequence (primers 1–4 in Figure 4B). For PCR reaction mixture and PCR conditions, refer to Before you begin steps 5d-e.

***Optional:*** The *pax-GFP* flanked by *loxP* sites can be excised through Cre expression (Figure 4B). This can improve expression of the integrated fragment. Cre recombinase lines such as BL#766 and BL#851 from the Bloomington stock centre can be used.

***Optional:*** Reintegration vectors can be introduced via the *attP* site via another round of injection. Examples include, introducing a GAL4 coding sequence into the locus, or reintroducing the CDS of the gene of interest but with a HA-tagged inserted within the CDS (Baena-Lopez et al., 2013, Poernbacher et al., 2019). (Figure 4C) Alternatively, instead of creating a conditional knockout, the re-integration vector can introduce another FRT cassette containing a mutant or tagged form of the gene of interest, thus generating a conditional switch allele. Details of the crossing scheme for the PhiC31-mediated reintegration can be found at (Hoppe and Ashe, 2021).

## Expected outcomes

This protocol creates a conditional knockout allele of the gene of interest. In Yu et al., 2020, a conditional *DWnt4* knockout allele was generated following this protocol. Tissue-specific gene inactivation (excision of the FRT-flanked DNA) was subsequently achieved by expressing a *UAS-Flp* transgene under the control of a wing-specific driver, thus creating a *DWnt4* mutant wing in an otherwise wild type animal.

## Limitations

Even though the FRT cassette, *attP* landing site and the selection markers are inserted into non-conserved regions of the genome, there is still a risk that reintegration of the rescue plasmid may not completely restore gene function. For essential genes, this can easily be assessed since the engineered flies would not be viable as homozygotes. Removal of the *pax-GFP* selection marker via Cre-expression could potentially solve the problem if the marker is affecting gene expression.

## Troubleshooting

### Problem 1

The target site search returned with no results on the CRISPR target finder tool, or only sites with multiple off-target sites (Before you begin step 4).

### Potential solution

Try ‘Low Stringency’ instead, with the caveat that off-target events will be more likely.

The guide length can be reduced to 17–18nt, as it has been suggested that shorter gRNAs can decrease undesired off-target events without sacrificing on-target editing efficiency (Fu et al., 2014).

### Problem 2

No unique restriction sites are present in MCS1 or MCS2 (Before you begin step 7).

### Potential solution

Restriction enzymes that produce compatible cohesive or blunt ends could also be used. More information can be found at https://international.neb.com/tools-and-resources/selection-charts/compatible-cohesive-ends-and-generation-of-new-restriction-sites.

### Problem 3

Gibson assembly of the 5′arm and FRT cassette into the parent repair plasmid was not successful, i.e., no colonies or getting a lot of background (Step-by-step Method step 11c)

### Potential solution

Make sure the vector is digested properly, run an uncut plasmid in the gel electrophoresis along with the cut plasmid, they should migrate differently in the gel.

Instead of Gibson Assembly, the components can be sequentially cloned into the repair vector using restriction sites from MCS1 via ligation.

### Problem 4

No GFP-positive candidates can be recovered from the progeny of injected individuals (Step-by-step Method step 16).

### Potential solution

Double check there are no SNPs in the chosen target sites, specifically in the gDNA of the Cas9-expressing strains used for injection.

Cutting efficiency of sgRNAs can be predicted using the online tool at https://www.flyrnai.org/evaluateCrispr/.

Other ways of assessing cutting efficiency include testing in S2R+ cells and using a T7 endonuclease assay (Viswanatha et al., 2018).

Make sure the DNA plasmids for injection are good quality, for example, measure absorbance from 230 nm to 320 nm, the A_260_/A_280_ ratio should be around 1.7–2.0. If not, re-transform and Maxi-prep the plasmid. Briefly centrifuge the DNA plasmids at top speed for several minutes prior to injection.

Most commercial injection services inject around 200–300 embryos. For difficult or inaccessible CRISPR loci injecting more embryos may be required to increase the chances of obtaining a successful transformant.

Alternatively, choose new CRISPR target sites.

### Problem 5

A range of *pax-GFP* signal intensities is seen in different candidate knockins (Step-by-step Method step 16).

### Potential solution

Occasionally an extra *pax-GFP* cassette gets inserted in the genome, with commensurate increased GFP signal in the eyes. Establish stable strain from a single candidate that does not have a brighter than average GFP signal and confirm proper location by PCR sequencing.

## Resource availability

### Lead contact

Further information and requests for resources and reagents should be directed to and will be fulfilled by the lead contact, Jean-Paul Vincent (jpvincent@crick.ac.uk).

### Materials availability

This study did not generate any unique reagents.

### Data and code availability

This study did not generate any unique datasets or codes.
